# Exploring the Feasibility of Deploying Future SDR Applications on RFSoC FPGA Device

**DOI:** 10.3390/s26144588

**Published:** 2026-07-20

**Authors:** Yuqin Zhao, Tiantai Deng, Edward Andrew Ball, Rola Saad

**Affiliations:** 1 Electronic and Electrical Engineering, The University of Sheffield, Mappin Building, Sheffield S1 3JD, UK; yzhao229@sheffield.ac.uk (Y.Z.); t.deng@sheffield.ac.uk (T.D.); e.a.ball@sheffield.ac.uk (E.A.B.); 2 Medical Physics and Biomedical Engineering, University College London, Gower Street, London WC1E 6BT, UK

**Keywords:** software-defined radio, FPGA, hardware acceleration, communication system, communication, 5G, 6G

## Abstract

Software-defined radio (SDR) has become a critical technology in modern communications, offering the flexibility and programmability required for dynamic signal processing. As the demand for high-performance SDR systems continues to increase, selecting an appropriate hardware platform has become increasingly important. This paper investigates the implementation of a state-of-the-art Chessboard-based Automatic Modulation Classification (CAMC) algorithm on a Field-Programmable Gate Array (FPGA)-based RFSoC platform, exploring various parallel datapath architectures and their impact on performance. The relationships between maximum operating frequency, bandwidth, and resource consumption are evaluated as the number of parallel instances and peripheral configurations are varied. The results show that the parallel CAMC design, incorporating all required peripherals, achieves a bandwidth of 29.0 GBps while consuming 82.31% of lookup tables (LUTs) and 50.67% of flip-flops (FFs), whereas the datapath-only design achieves a bandwidth of 24.8 GBps with significantly lower resource consumption. In addition, as the number of parallel instances increases, the number of High-Performance (HP) ports or master ports increases, the maximum operating frequency decreases, and bandwidth growth gradually saturates. The presented implementation study and open-source hardware designs provide practical reference architectures for evaluating the trade-offs between bandwidth, performance, and resource utilisation in FPGA-based SDR systems.

## 1. Introduction

Software-defined radio (SDR) plays a pivotal role in modern communications by enabling flexible, programmable signal processing systems [1]. SDR systems, typically implemented on platforms such as general-purpose processors (GPPs) or field-programmable gate arrays (FPGAs), offer extensive configurability, including adjustable frequency, bandwidth, modulation schemes, and data rates [1]. Over the years, communication systems have evolved significantly: 2G supported data rates of 9.6 kbps, 2G+ (EDGE) reached 384 kbps, 3G offered 2 Mbps, 4G enabled 1 Gbps, and 5G achieved up to 20 Gbps [2,3,4,5,6]. SDR has already proven essential in 4G and 5G communication systems, providing the flexibility required for these high-performance networks [7,8]. Looking ahead, future wireless systems are expected to support increasingly demanding workloads, including AI-native communications, integrated sensing and communication, and massive MIMO processing, which will place even greater demands on SDR implementations [9,10]. Consequently, it is increasingly important to investigate scalable FPGA-based SDR implementations that can meet these growing performance requirements.

To improve SDR system performance, selecting an appropriate hardware platform is essential. SDR systems have been deployed on a variety of platforms, including GPPs, FPGAs, hybrid FPGA+GPP architectures, and application-specific integrated circuits (ASICs) [11]. While GPPs are favoured for their high programmability, this flexibility often compromises performance and power efficiency [12]. In contrast, ASICs offer superior performance and energy efficiency but lack the adaptability required for diverse applications [12]. Modern FPGA platforms, frequently integrated with ARM processors, provide a balanced solution—offering better performance and power efficiency than GPPs while maintaining sufficient programmability to support flexible SDR designs. By leveraging the inherent parallelism of FPGAs, these platforms also deliver high throughput [11,12]. Furthermore, the inherent reconfigurability and high bandwidth of FPGAs make them a compelling choice for future SDR systems, such as those envisioned for 6G sensing and communication [13,14,15]. Their combination of strong performance, power efficiency, fast design turnaround, and the ability to process large volumes of real-time data in parallel further highlights their suitability for these high-demand applications [11,12].

In this study, we implement the state-of-the-art SDR application, Chessboard-based Automatic Modulation Classification (CAMC) [16,17], on an FPGA platform together with all required peripheral components. Although CAMC is an automatic modulation classification algorithm rather than a complete 6G sensing application, its core computation consists primarily of multiply-and-accumulate (MAC) operations, which are also fundamental to many wireless baseband signal-processing functions, including finite impulse response (FIR) filtering, infinite impulse response (IIR) filtering, fast Fourier transform (FFT), and multiple-input multiple-output (MIMO) processing [18,19,20]. Therefore, CAMC is adopted in this work as a representative compute-intensive SDR workload for investigating the scalability of FPGA-based SDR implementations. To systematically explore implementation scalability, multiple parallel hardware architectures are developed to investigate the relationships among maximum operating frequency, bandwidth, and resource utilisation as the number of parallel CAMC instances increases. In addition, a datapath-only architecture is implemented under the same platform for comparison, providing practical reference designs for evaluating bandwidth–performance–resource trade-offs in FPGA-based SDR systems.

The key results of this research are summarised as follows:

1. This work presents a systematic implementation study of the CAMC SDR application on the RFSoC 4x2 platform, investigating how bandwidth, maximum operating frequency, and resource utilisation scale with increasing parallelism. The results provide practical guidance for SDR researchers targeting FPGA-based implementations.
2. The parallel CAMC design, including all required peripherals, achieves a bandwidth of 29.0 GBps while utilising 82.31% of LUTs and 50.67% of flip-flops (FFs). In comparison, the datapath-only design achieves a bandwidth of 24.8 GBps while consuming only 4.61% of LUTs and 3.43% of FFs.
3. Experimental results show that increasing the number of parallel instances, HP ports, and master ports improves bandwidth but also reduces the maximum operating frequency and increases FPGA resource utilisation, illustrating the practical trade-offs involved in scaling FPGA-based SDR systems.
4. The developed hardware designs are released as open-source reference implementations, allowing researchers to replace the CAMC accelerator with their own SDR kernels and evaluate bandwidth, performance, and resource utilisation under the same RFSoC platform.

## 2. Methodology and Hardware Structure

This section presents the development of several hardware architectures based on FPGAs. The Automatic Modulation Classification (AMC) module, a crucial application in Software-Defined Radio (SDR) aimed at enhancing system efficiency through the identification of modulation types, has been selected as the target module. The Chessboard-based Automatic Modulation Classification (CAMC) algorithm has been chosen as the representative example because its hardware implementation has previously demonstrated competitive classification performance together with efficient FPGA resource utilisation [16,17]. Furthermore, the core computation of CAMC is multiply-and-accumulate (MAC), which is also a fundamental operation in many wireless baseband signal-processing functions, including finite impulse response (FIR) filtering, infinite impulse response (IIR) filtering, fast Fourier transform (FFT), and multiple-input multiple-output (MIMO) processing [18,19,20]. Therefore, CAMC is adopted as a representative MAC-intensive SDR workload for investigating the scalability of FPGA-based SDR implementations rather than as a direct implementation of a specific 6G sensing application. Additionally, a datapath-only hardware architecture has been implemented using Direct Memory Access (DMA) to emulate a data-transfer-only function.

**Figure 1:**
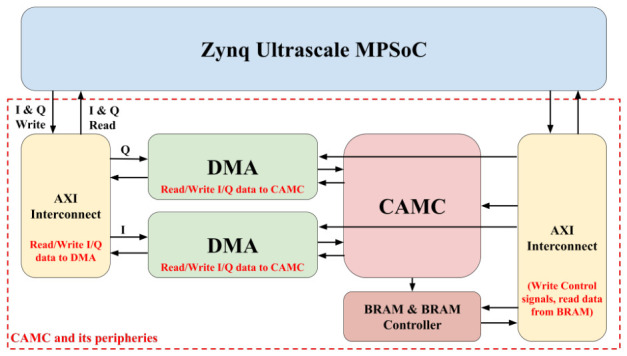
Hardware Structure of CAMC.

Figure 1 illustrates the hardware architecture of the CAMC module, along with its associated peripherals. To implement the full SDR functionality, peripheral modules such as the BRAM Controller, BRAM Generator, DMA, and AXI Interconnect are utilised. The I/Q data is initially transmitted to the CAMC FPGA hardware from a High-Performance (HP) port of the Zynq UltraScale+ MPSoC via the AXI Interconnect and DMA, employing the AXI Stream protocol to achieve high transfer speeds. Once the data is received from the DMA, the CAMC module processes the algorithm within the FPGA fabric. The processed data is then stored directly in the BRAM on the FPGA. Finally, the Zynq UltraScale+ MPSoC reads the results from BRAM through the AXI Interconnect and BRAM Controller and subsequently displays the results on a PC. All AXI Stream ports are connected to the HP ports to achieve high-speed data transmission between the MPSoC and FPGA, while all IP cores are connected to the master ports to enable proper control by the MPSoC.

The HP ports serve as the primary interface for high-throughput data transfer between the programmable logic (PL) and DDR memory. Their performance depends on both deterministic traffic from PL masters and non-deterministic traffic from processor-driven software. By providing configurable controls, HP ports allow designers to shape memory access patterns, enabling efficient utilisation of DDR bandwidth and supporting high-performance system operation [21].

**Figure 2:**
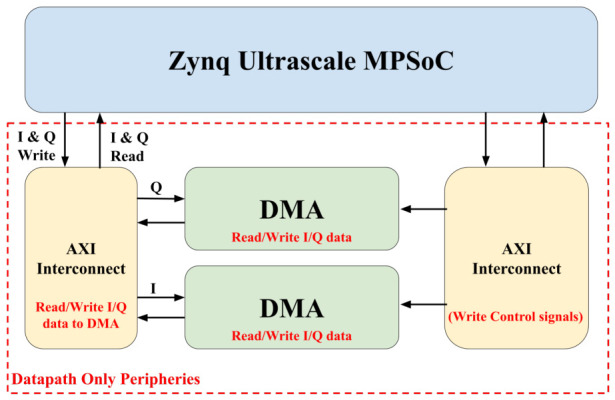
Hardware Structure of Data Path (Using DMA).

Figure 2 illustrates the datapath-only hardware architecture, which utilises only DMA and the AXI Interconnect. In this architecture, the I/Q data is first transferred from a High-Performance (HP) port of the Zynq UltraScale+ MPSoC through the AXI Interconnect to the DMA, and then returned. The AXI Interconnect on the right serves as the controller for the DMA modules. This design serves as a reference framework that allows users to estimate the available FPGA resources and bandwidth when integrating their own SDR hardware accelerators.

**Figure 3:**
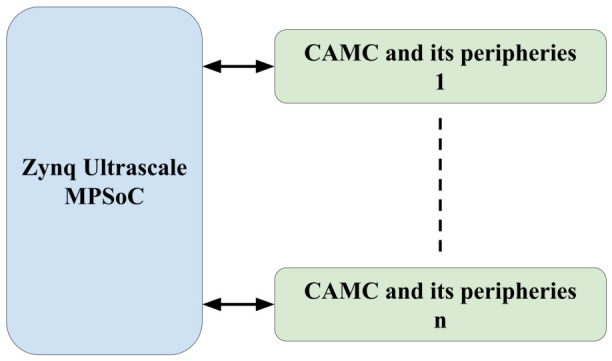
Parallel Hardware Structure of CAMC and its peripheries.

**Figure 4:**
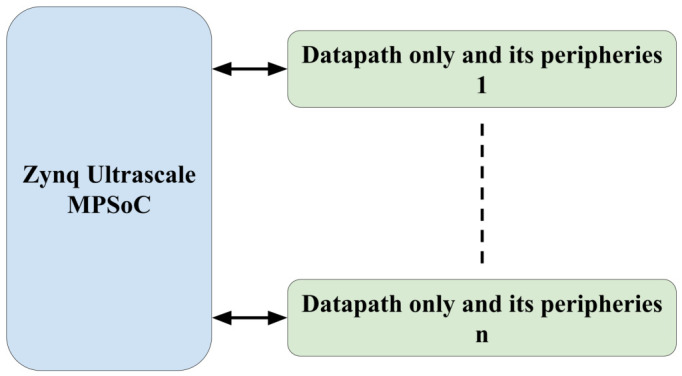
Parallel Hardware Structure of Data Path (Using DMA).

Figure 3 and Figure 4 illustrate the parallel hardware designs for the CAMC and datapath-only architectures, respectively. The “CAMC and its peripherals” architecture is consistent with the design shown in Figure 1, while the “Datapath-only and its peripherals” corresponds to the architecture depicted in Figure 2. To investigate the relationship between bandwidth and resource consumption, the number of parallel instances is incrementally increased. These two hardware implementations serve as reference designs for evaluating the scalability of FPGA-based SDR systems and for exploring the trade-offs between bandwidth, operating frequency, and resource utilisation under increasing levels of parallelism.

All hardware architectures presented in this section were synthesised and implemented using Vivado [22]. The resulting bandwidth, maximum operating frequency, and resource utilisation are analysed and discussed in the following section.

## 3. Results and Comparisons

The SDR system is synthesised and implemented using Vivado 2024.1, targeting the RFSoC 4x2 platform (XCZU48DR-1FFVG1517E).

The CAMC-based SDR application [16,17], initially developed using Vivado HLS 2019, has been updated and resynthesised with Vivado HLS 2024. This updated version generates the hardware design from its high-level C/C++ algorithm. The single CAMC hardware design consumes 2492 LUT, 2765 FF, 20.5 BRAM, and 30 DSPs.

As described earlier, two designs are synthesised and implemented in Vivado: (1) a comprehensive SDR application that integrates the CAMC algorithm along with its peripheral modules, and (2) a standalone datapath hardware design for the SDR platform. These designs are evaluated in terms of bandwidth, maximum operating frequency, and resource utilisation.

The bandwidth (for either I or Q) is calculated using the equation provided in Equation (1) below:

(1) bandwidth(GBps)=(max_FPGA_freq∗no_DMA∗DMA_bit)/8

In the FPGA hardware design, bandwidth represents the maximum amount of data, in bits or bytes, that can be processed per second. It should be noted that Equation (1) estimates the theoretical peak bandwidth of the hardware implementation under the assumptions of continuous data streaming, full DMA utilisation, and ideal data transfer. In practice, the achievable throughput may be affected by AXI protocol overhead, memory arbitration, DMA scheduling latency, and software interaction. Therefore, the calculated bandwidth is used as a consistent implementation-level metric for comparing different hardware configurations rather than as a measurement of sustained application throughput.

In reference to Equation (1), *max_FPGA_freq* can be calculated from the worst slack time provided by the Vivado tool, which is used to determine the maximum operating frequency achievable by the FPGA implementation. *No_DMA*, on the other hand, represents the number of I/Q channels implemented in the current hardware design. *DMA_bit* indicates the number of bits that can be transmitted in each clock cycle. Thus, by calculating all three mentioned data points, the bandwidth—the number of bytes (by division of 8) that can be transmitted in one second—can be determined. In the CAMC parallel hardware design, the number of DMAs represents the total number of I and Q channels, and the number of parallel CAMC instances is half the number of DMAs. Similarly, in the datapath-only hardware design, the number of DMAs also represents the total number of I and Q channels, and the number of parallel transmission designs is half the number of DMAs.

### 3.1. Bandwidth of SDR System with Peripheries

In this subsection, we present the results based on the CAMC SDR application, including all associated peripheral components—such as the MPSoC, DMA, BRAM, and AXI interconnect.

**Figure 5:**
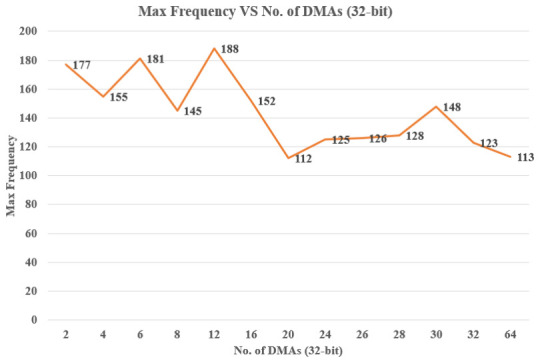
The Trend of Max Frequency vs. the number of DMAs (32-bit).

**Figure 6:**
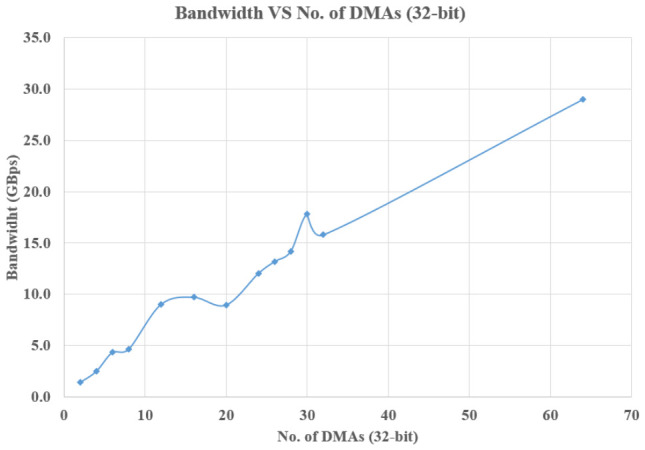
The Trend of Bandwidth vs. the number of DMAs (32-bit).

Table 1 illustrates the relationship between the number of DMAs (equivalent to the number of active I or Q channels), the maximum operating frequency of the entire hardware system, and the corresponding bandwidth in GBps. To clearly visualise these trends, Figure 5 and Figure 6 depict the relationship between the number of DMAs and both the maximum frequency and bandwidth, respectively.

Overall, the bandwidth of 29.0 GBps can be reached when 64 32-bit DMAs are utilised, operating at an FPGA frequency of 113 MHz (which corresponds to the maximum frequency that the algorithm can be run on the FPGA). At this configuration, resource utilisation is 82.31% for LUTs and 50.67% for flip-flops (FFs). For the maximum resource-consuming hardware design, 32 parallel CAMC instances with 64 DMAs, if additional SDR applications in 6G communication, such as FFT, need to be implemented after CAMC, the remaining resources will be sufficient to support the implementation of 18 1024-point FFT (strided type) hardware modules, as published in [23]. If FFT needs to be implemented alongside 16 parallel CAMC instances with 32 DMAs, it can be implemented with up to 60 FFT modules. These results indicate that the RFSoC platform provides sufficient resources to support additional signal-processing modules, such as FFT accelerators, while maintaining relatively high bandwidth, illustrating the trade-off between achievable bandwidth and available FPGA resources.

Figure 5 shows that the maximum operating frequency of the SDR system decreases as the number of DMAs (or I/Q channels) increases. Figure 5 shows that the maximum operating frequency of the SDR system generally decreases as the number of DMAs (or I/Q channels) increases. The non-monotonic frequency variations observed for intermediate configurations are mainly attributed to the heuristic nature of FPGA placement and routing. Differences in routing congestion, placement decisions, and timing optimisation can produce slightly different timing results for hardware implementations of similar complexity, even when generated using the same implementation flow.

**Figure 7:**
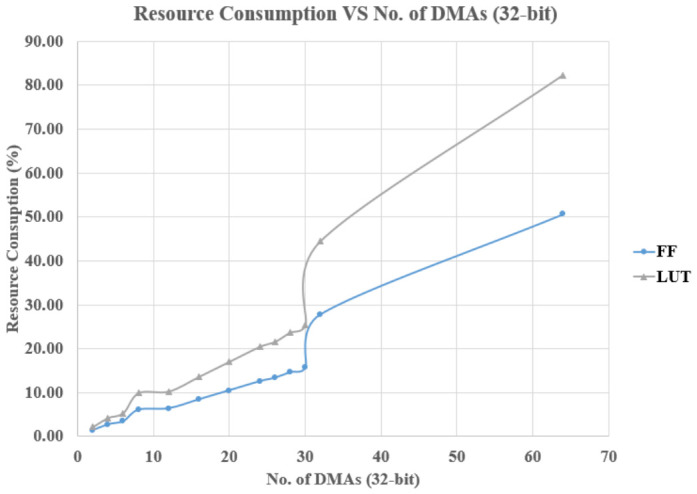
The Trend of Resource Consumption vs. the number of DMAs (32-bit).

**Figure 8:**
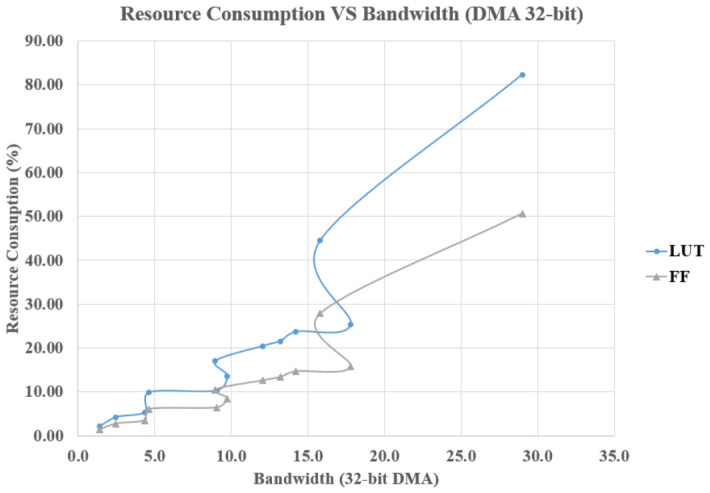
The Trend of Resource Consumption vs. Bandwidth (DMA 32-bit).

Figure 6 presents the trend of bandwidth versus the number of DMAs. A steep increase in bandwidth is observed when only a small number of DMAs are utilised. However, as the number of DMAs increases, the rate of bandwidth increase begins to slow down and even declines slightly beyond 20 and 30 DMAs. This is mainly due to the decrease in maximum operating frequency, which offsets the gains from additional DMAs, leading to a diminishing bandwidth trend. Table 2 and Figure 7 illustrate the relationship between the number of DMAs and resource utilisation (LUTs and FFs). Figure 8 further demonstrates how bandwidth correlates with resource consumption. Based on this information, the required bandwidth and the corresponding number of DMAs can be determined, as well as the associated resource consumption on the RFSoC. Notably, a 30-DMA implementation provides the optimal balance of bandwidth and resource usage, since resource consumption increases significantly beyond 30 DMAs. Although the 32-DMA configuration provides additional parallelism, it requires three master ports instead of one, resulting in a substantial increase in routing complexity and FPGA resource utilisation. The additional routing overhead reduces the achievable operating frequency from 148 MHz to 123 MHz, outweighing the bandwidth gain obtained from the two additional DMA channels. Consequently, the measured peak bandwidth decreases from 17.8 GBps to 15.8 GBps, making the 30-DMA configuration a more resource-efficient operating point for the investigated hardware architecture.

The data indicates that resource consumption increases significantly as the number of DMAs approaches 30 or when the bandwidth reaches approximately 17.8 GBps. Beyond this point, resource utilisation increases sharply because additional HP and master ports are required. Resource usage then remains relatively stable between the 32- and 64-DMA configurations because the number of HP and master ports remains unchanged while only additional CAMC instances are added.

From the experiments, for fewer than 32 DMAs, the design uses a single master port, while 3 master ports are required for designs with 32 and 64 DMAs. This increase in the number of master ports is the primary reason for the rise in resource consumption. Since the number of HP and master ports remains constant between 32 and 64 DMAs, adding more DMAs does not significantly affect overall resource usage within this range.

**Figure 9:**
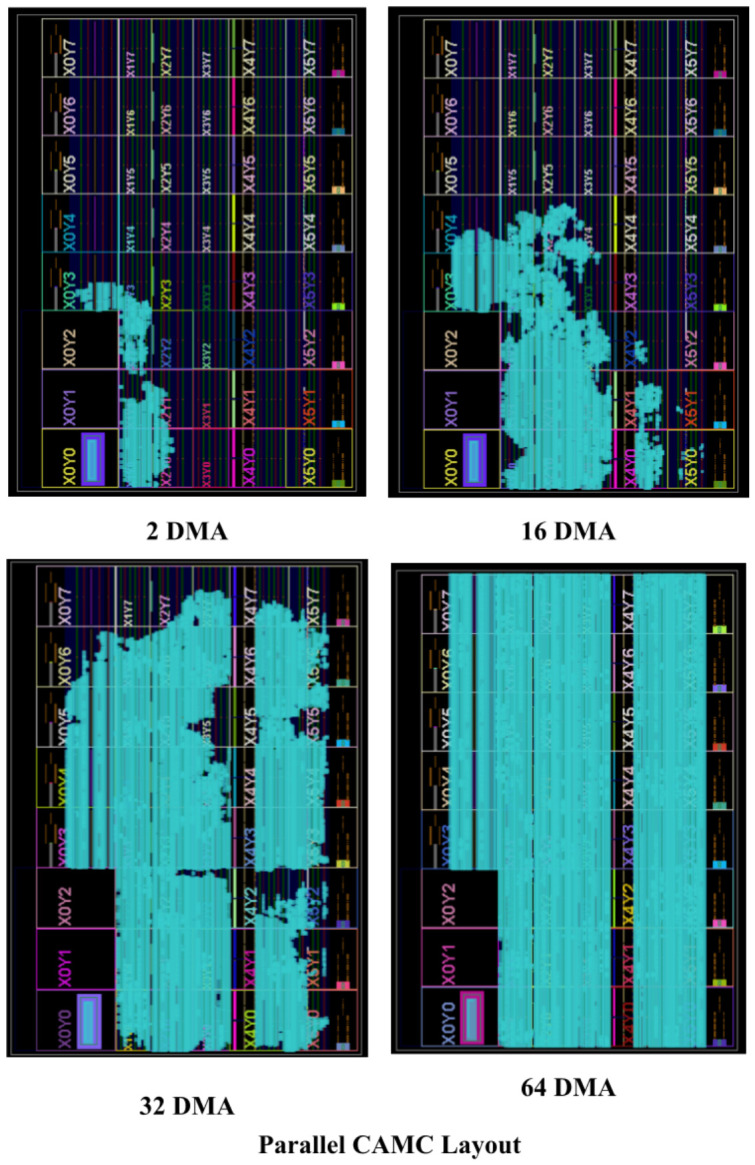
Layout of Parallel CAMC Hardware Implementation (DMA 32-bit).

**Figure 10:**
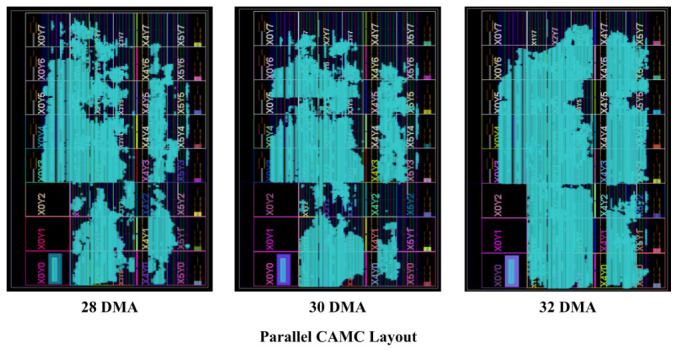
Comparison between Layouts of Parallel CAMC Hardware Implementation (DMA 32-bit) of 28, 30, 32 DMAs.

The layouts of the hardware implementation of CAMC with 2 DMAs, 16 DMAs, 32 DMAs, and 64 DMAs in Vivado are shown in Figure 9. As shown in Figure 9 and Figure 10, increasing the design from 30 to 32 DMAs results in a 17% increase in LUTs and a 12% increase in FFs, which is considerably larger than the modest overhead observed when scaling from 28 to 30 DMAs (approximately 2% LUTs and 1% FFs). In contrast, the design with 64 DMAs exhibits a substantial rise in resource usage, consuming over 80% of LUTs and more than 50% of FFs, thereby utilising the majority of the available hardware resources on RFSoC. This increase in resource consumption is attributed to the additional master ports and HP ports required for the design. Therefore, a trade-off exists between the complexity of SDR applications and achievable bandwidth. For instance, to reach the maximum bandwidth on an RFSoC platform, the resources remaining for implementing additional complex SDR modules are limited. Conversely, incorporating more complex SDR applications can further restrict the achievable bandwidth due to increased resource consumption.

**Figure 11:**
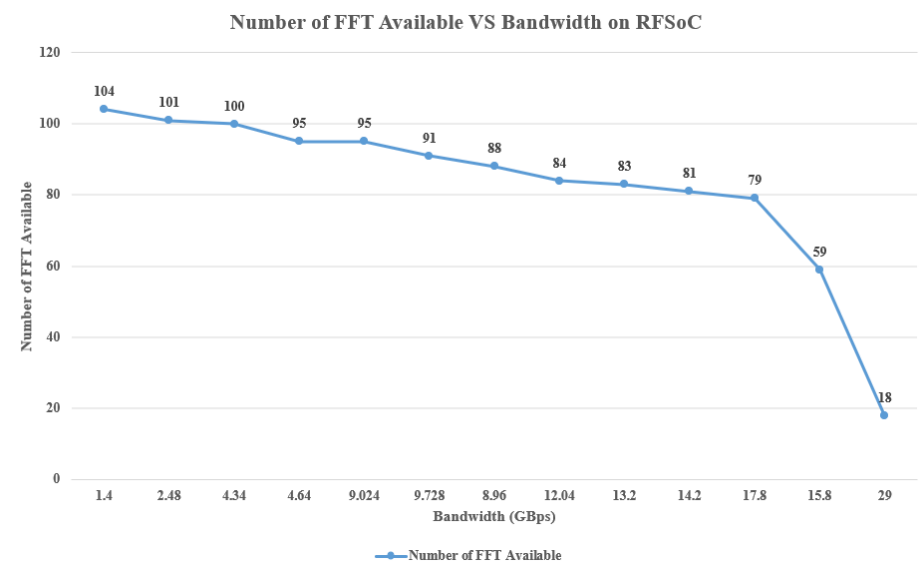
Number of Available FFT vs. Bandwidth.

Figure 11 shows the trend of the number of FFTs that can be implemented based on the remaining available resources after deploying the parallel CAMC design and its peripherals, plotted against bandwidth. The figure reveals a steady decrease in the number of FFTs up to 17.8 GBps, which corresponds to the use of 30 DMAs. However, when more than 32 DMAs are utilised, additional master and HP ports are required, leading to substantial resource consumption. As a result, the number of FFTs that can be implemented on the RFSoC decreases sharply due to the limited remaining resources. Therefore, the 30-DMA configuration represents a practical operating point that provides a favourable balance between bandwidth and FPGA resource utilisation for the investigated hardware architecture. This is also consistent with the observed reduction in bandwidth from 17.8 GBps (30 DMAs) to 15.8 GBps (32 DMAs), where the additional routing and control overhead outweigh the benefit of the extra DMA channels.

### 3.2. Bandwidth of Data Path Only

In this section, we evaluate a hardware design that solely implements the dataflow architecture of the SDR system to analyse the relationship between the number of DMAs, bandwidth, and resource consumption. The number of DMAs varies from 1 to 8, corresponding to the maximum number of high-performance (HP) ports available for use in this SDR design. Each DMA processes data at a width of 128 bits, the maximum supported in the DMA setup. The results of this implementation provide a baseline for estimating the bandwidth and FPGA resources required by the SDR datapath alone, allowing users to better assess the additional overhead introduced when integrating their own SDR processing modules.

The highest achievable bandwidth in this configuration is 24.8 GBps, obtained with eight 128-bit DMAs operating at a frequency of 194 MHz. At this point, the design consumes 4.61% of LUTs and 3.42% of flip-flops (FFs). Compared to the full CAMC-based SDR implementation, the datapath-only design achieves substantially lower resource consumption and a higher operating frequency. It should be noted that this comparison reflects two different practical hardware configurations, including different DMA interface widths (32-bit for CAMC and 128-bit for the datapath-only design), and therefore is intended to illustrate overall implementation characteristics rather than isolate the overhead of individual hardware components.

**Figure 12:**
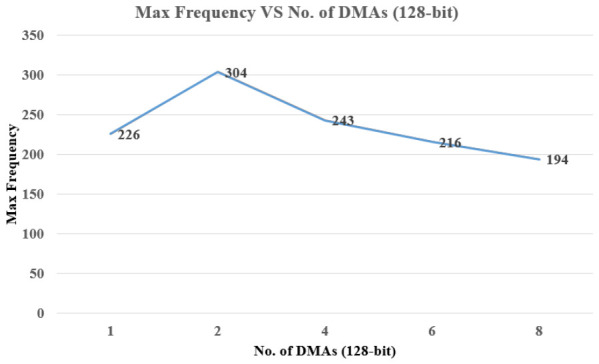
The Trend of Max Frequency vs. the number of DMAs (128-bit).

**Figure 13:**
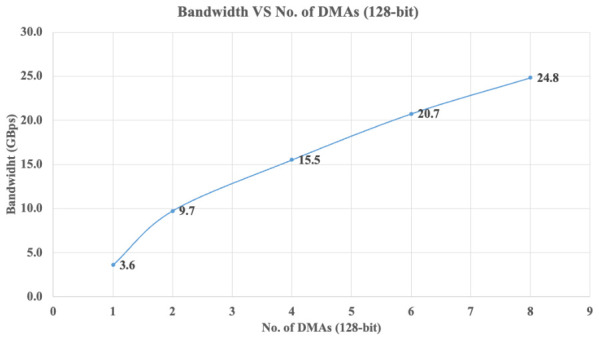
The Trend of Bandwidth vs. the number of DMAs (128-bit).

Table 3 shows the relationship between the number of DMAs (equivalent to the number of active I/Q channels), the maximum operating frequency of the hardware system, and the resulting bandwidth in GBps. Figure 12 and Figure 13 further illustrate the trends in frequency and bandwidth as the number of DMAs increases.

Figure 12 demonstrates that the maximum hardware design operating frequency initially increases, peaking at 204 MHz when two 128-bit DMAs are used. After this peak, the frequency gradually decreases as more DMAs are added.

Figure 13 illustrates a sharp increase in bandwidth when two 128-bit DMAs are used, corresponding to the frequency peak mentioned above. However, as the number of DMAs exceeds two, the bandwidth growth slows down. This trend is primarily due to the decrease in operating frequency, which reduces the overall bandwidth gain from adding more DMAs.

**Figure 14:**
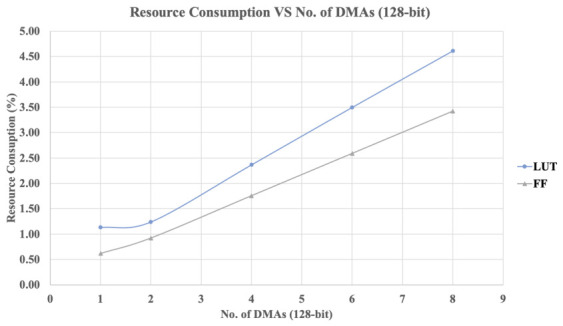
The Trend of Resource Consumption vs. the number of DMAs (128-bit).

**Figure 15:**
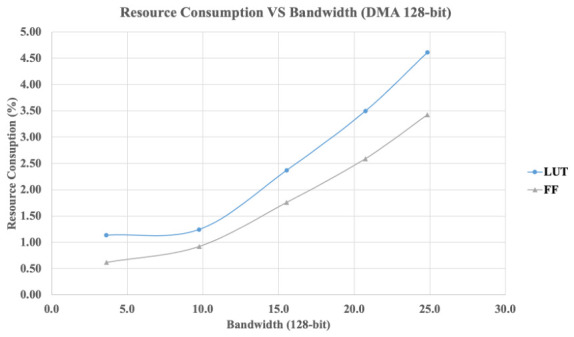
The Trend of Resource Consumption vs. Bandwidth (DMA 128-bit).

Table 4 and Figure 14 present the relationship between the number of DMAs and resource utilization in terms of LUTs and FFs. Figure 15 further illustrates how resource consumption scales with bandwidth. Overall, the observed resource usage is primarily attributed to the combined impact of DMA implementations and MPSoC-related overhead.

The results indicate that resource utilisation increases only slightly up to two DMAs, or approximately 9.7 GBps in bandwidth. Beyond this point, resource consumption rises more significantly. This behaviour is primarily attributed to the increasing utilisation of the available HP ports and the associated routing complexity, which gradually limits further improvements in implementation scalability.

In summary, both the full SDR system hardware (including the CAMC module and its peripheral components) and the SDR dataflow-only hardware follow a consistent trend: the rate of bandwidth increase becomes saturated as the number of active DMAs (I/Q channels) increases. This limitation is primarily due to the rise in resource utilisation and the decrease in the maximum operating frequency of the hardware.

Resource consumption is significantly influenced by 2 key factors:

- The number of master ports utilised, which substantially impacts resource consumption.
- The number of high-performance (HP) ports utilised, which directly impacts logic complexity and routing.

A clear pattern emerges: resource usage increases with the number of HP ports in use, but this growth begins to flatten as the number of DMAs rises without a corresponding increase in HP port count.

Furthermore, the analysis suggests a correlation between lower resource utilisation and higher operating frequencies. This is evident from the frequency-resource comparison between the full SDR system and the dataflow-only designs, as well as the trends relating resource consumption to the number of DMAs.

If readers aim to achieve the highest possible bandwidth without heavily consuming other IPs such as BRAM, additional SDR applications, or power, they can deploy as many DMAs as the platform allows to maximise bandwidth. However, for users who need to reserve resources for other SDR applications or prefer a more bandwidth-to-resource-efficient design, the number of DMAs should be limited to fewer than 30 to avoid excessive resource overhead caused by master ports and HP ports, based on our CAMC parallel hardware design.

## 4. Open-Source Hardware Design for Evaluation

Three hardware designs of CAMC with its peripherals have been uploaded to GitHub (https://github.com/RainChinChao/RFSoC_Guide.git, accessed on 1 June 2026), featuring 2, 30, and 64 DMAs. The 2 DMA design represents the minimum requirements for an SDR implementation, with one DMA for I-channel transmission and another for Q-channel transmission. The 30-DMA design represents a balanced “sweet spot” between bandwidth and resource consumption. Designs exceeding 30 DMAs require additional HP ports and master ports, resulting in a significant increase in resource usage. The 64-DMA design targets users with extremely high bandwidth requirements.

**Figure 16:**
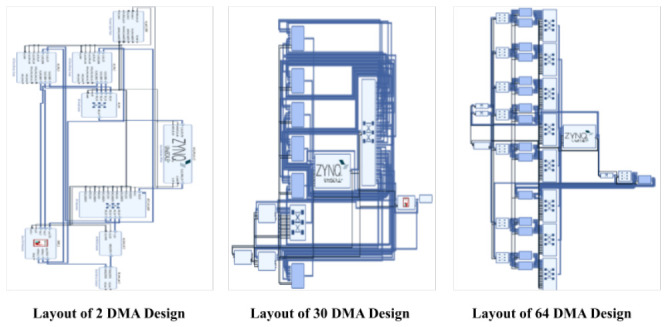
Hardware Architecture Layout of 2, 30, and 64 DMA Designs.

**Figure 17:**
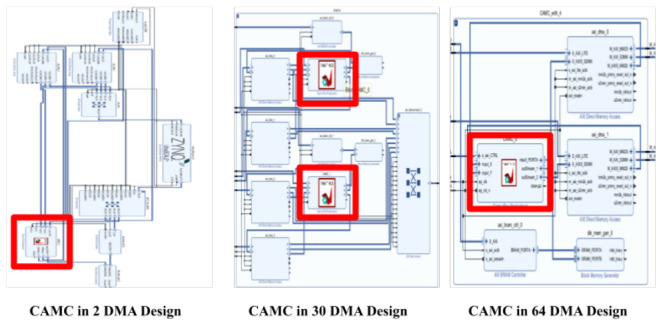
CAMC in Designs with 2, 30, and 64 DMA.

On GitHub, users can find three folders named CAMC_32_2DMA, CAMC_32_30DMA, and CAMC_32_64DMA. These designs can be easily downloaded and opened in Vivado using the Block Design function. The layouts of the hardware architectures for the three designs are shown in Figure 16. For the 30 and 64 DMA designs, CAMC is placed hierarchically to simplify layout and ease placement. Users can replace the CAMC module with their own Vivado HLS-generated design, provided it includes two AXI-Stream ports, a BRAM port, and a set of AXI-Lite control ports, as illustrated in Figure 17. Synthesis and implementation in Vivado can then be run to estimate resource usage and performance.

Based on the provided open-source hardware designs, users can experimentally integrate their own designs into the existing hardware architecture to estimate performance, bandwidth, resource consumption, and the trade-offs between these metrics after running synthesis and implementation.

## 5. Conclusions and Future Work

This paper investigated the relationship between maximum operating frequency, bandwidth, and resource consumption when implementing the Chessboard-based Automatic Modulation Classification (CAMC) algorithm and a datapath-only SDR architecture on an RFSoC FPGA platform. As CAMC is a representative MAC-intensive SDR workload, the presented hardware implementations provide a practical case study for evaluating the scalability of FPGA-based SDR architectures. The experimental results provide practical guidance for selecting the number of DMA channels and understanding the trade-offs between achievable bandwidth, operating frequency, and FPGA resource utilisation.

The parallel CAMC design, including all required peripheral modules, achieves a bandwidth of 29.0 GBps while consuming 82.31% of LUTs and 50.67% of flip-flops (FFs). In contrast, the datapath-only design achieves a bandwidth of 24.8 GBps while consuming only 4.61% of LUTs and 3.43% of FFs. The differences between the two implementations are influenced by several factors, including the additional peripheral modules required by the complete SDR system and the different DMA interface widths (32-bit for the CAMC implementation and 128-bit for the datapath-only implementation). Consequently, the comparison represents two practical implementation scenarios rather than an isolated evaluation of peripheral overhead.

For the parallel CAMC implementation, the maximum operating frequency generally decreases as the number of parallel instances, HP ports, and master ports increases. Consequently, the bandwidth improvement gradually saturates while FPGA resource utilisation increases more rapidly. Experimental results further indicate that the 30-DMA configuration provides a favourable balance between bandwidth and resource utilisation for the investigated hardware architecture, whereas additional master ports required beyond this point introduce increased routing complexity and timing degradation.

For the datapath-only implementation, the maximum operating frequency reaches up to 304 MHz when two DMA channels are used. Beyond this point, bandwidth continues to increase with additional DMA channels, although the rate of improvement gradually decreases as operating frequency reduces and FPGA resource utilisation increases.

Finally, three open-source hardware reference designs are provided on GitHub, enabling users to replace the CAMC accelerator with their own SDR processing modules and evaluate bandwidth, performance, and FPGA resource utilisation using the same RFSoC platform. These reference designs provide a practical framework for exploring implementation trade-offs in FPGA-based SDR systems.

## Figures and Tables

**Table 1:** Hardware Bandwidth vs. the number of DMA (32-bit) utilised.

No. DMAs	Max F (MHz)	Bandwidth (I&Q in Total) (GBps)
2	177	1.4
4	155	2.5
6	181	4.3
8	145	4.6
12	188	9.0
16	152	9.7
20	112	9.0
24	125	12.0
26	126	13.2
28	128	14.2
30	148	17.8
32	123	15.8
64	113	29.0

**Table 2:** Hardware Resource Consumption vs. the number of DMA (32-bit) utilised.

No. DMAs	LUT	FF
2	9205 (2.16%)	11,808 (1.39%)
4	17,941 (4.22%)	23,005 (2.70%)
6	22,734 (5.35%)	29,374 (3.45%)
8	42,066 (9.89%)	51,496 (6.05%)
12	43,599 (10.25%)	54,287 (6.38%)
16	57,848 (13.60%)	71,757 (8.44%)
20	72,433 (17.03%)	89,291 (10.50%)
24	86,854 (20.42%)	106,832 (12.56%)
26	91,653 (21.55%)	113,549 (13.35%)
28	100,719 (23.68%)	124,446 (14.63%)
30	107,853 (25.36%)	133,430 (15.69%)
32	189,228 (44.49%)	236,713 (27.83%)
64	350,060 (82.31%)	431,015 (50.67%)

**Table 3:** Hardware Bandwidth vs. the number of DMA (128-bit) utilised.

No. DMAs	Max F (MHz)	Bandwidth (I&Q in Total) (GBps)
1	226	3.6
2	304	9.7
4	243	15.5
6	216	20.7
8	194	24.8

**Table 4:** Hardware Resource Consumption vs. the number of DMA (128-bit) utilised.

No. DMAs	LUT	FF
1	4819 (1.13%)	5269 (0.62%)
2	5284 (1.24%)	7854 (0.92%)
4	10,065 (2.37%)	14,941 (1.76%)
6	14,873 (3.50%)	22,028 (2.59%)
8	19,601 (4.61%)	29,102 (3.42%)

## Data Availability

The original contributions presented in this study are included in the article. Further inquiries can be directed to the corresponding author.
